# Association Between a History of Dengue Fever and the Risk of Systemic Autoimmune Rheumatic Diseases: A Nationwide, Population-Based Case-Control Study

**DOI:** 10.3389/fmed.2021.738291

**Published:** 2021-11-03

**Authors:** Yun-Wen Chen, Tsu-Yi Hsieh, Ching-Heng Lin, Hsian-Min Chen, Chi-Chien Lin, Hsin-Hua Chen

**Affiliations:** ^1^Division of Allergy, Immunology and Rheumatology, Department of Internal Medicine, Taichung Veterans General Hospital, Taichung City, Taiwan; ^2^Department of Medical Education, Taichung Veterans General Hospital, Taichung City, Taiwan; ^3^Ph.D. Program of Business, Feng Chia University, Taichung City, Taiwan; ^4^School of Medicine, National Yang-Ming University, Taipei City, Taiwan; ^5^Department of Industrial Engineering and Enterprise Information, Tunghai University, Taichung City, Taiwan; ^6^Department of Medical Research, Taichung Veterans General Hospital, Taichung City, Taiwan; ^7^Department of Healthcare Management, National Taipei University of Nursing and Health Sciences, Taipei City, Taiwan; ^8^Department of Public Health, College of Medicine, Fu Jen Catholic University, New Taipei City, Taiwan; ^9^Center for Quantitative Imaging in Medicine (CQUIM), Department of Medical Research, Taichung Veterans General Hospital, Taichung City, Taiwan; ^10^Department of Computer Science and Information Engineering, National United University, Miaoli City, Taiwan; ^11^Institute of Biomedical Science and Rong Hsing Research Center for Translational Medicine, Chung Hsing University, Taichung City, Taiwan

**Keywords:** dengue, history, systemic autoimmune rheumatic disease (SARD), risk, claims data

## Abstract

**Purpose:** To determine the association between a history of clinically diagnosed dengue infection and the risk of systemic autoimmune rheumatic diseases (SARDs).

**Methods:** Using claims data from the 1997–2013 Taiwanese National Health Insurance Research Database, we included 74,422 patients who were diagnosed with SARDs and 297,688 patients without SARDs who were matched (in a 1:4 ratio) for age, sex, year of SARDs index date, and city of residence. The associations between the development of SARDs and a history of dengue infection (International Classification of Diseases, Ninth Revision, Clinical Modification code 061) were investigated using conditional logistic regression analysis shown as odds ratios (ORs) with 95% confidence intervals (CIs) after adjusting for potential confounders.

**Results:** We included 17,126 patients with systemic lupus erythematosus (SLE), 15,531 patients with Sjogren's syndrome (SS), 37,685 patients with rheumatoid arthritis (RA), 1,911 patients with systemic sclerosis (SSc), 1,277 patients with dermatomyositis (DM), and 892 patients with polymyositis (PM). SLE (OR, 4.55; 95% CI, 2.77–7.46; *p* <0.001) risk was significantly associated with a history of dengue infection. However, no statistically significant association was found between dengue infection and SS (OR, 1.41; 95% CI, 0.88–2.26; *p* = 0.155), RA (OR, 1.03; 95% CI, 0.70–1.50; *p* = 0.888), SSc (OR, 1.97; 95% CI, 0.38–10.29; *p* = 0.420), DM (OR, 0.54; 95% CI, 0.04–7.27; *p* = 0.641), or PM (OR, 2.08; 95% CI, 0.23–18.79; *p* = 0.513).

**Conclusion:** This study revealed that a history of dengue infection was significantly associated with the risk of SLE, but not SS, RA, SSc, DM, or PM.

## Introduction

Systemic autoimmune rheumatic diseases (SARDs) develop when the immune system is dysregulated such that its tolerance to self-antigens is lost. The exact cause of SARDs has not yet been elucidated. However, it is known that genetics, infections, and environmental predisposing factors are all closely related. Dengue, a mosquito-borne disease, poses a global burden. Approximately 390 million infections and 96 million cases occur each year. The dengue virus (DENV) has four serotypes, DENV-1, DENV-2, DENV-3, and DENV-4. The clinical features of dengue infection include fever, spontaneous bleeding, and organ failure (1, 2). García G et al. reported that after dengue infection, patients may suffer from persistent myalgia or arthralgia and have alterations in some autoimmune markers such as the immune complex, which may be associated with autoimmune-based disturbance (3). DENV infection triggers several pathways, including DENV nonstructural protein 1 (NS1) antibody-induced autophagy, the activation of the complement pathway, the dysregulation of B and T cells, elevated levels of cytokines such as tumor necrosis factor (TNF)-α and interferon (IFN)-γ, which were considered to be associated with the development of autoimmunity in dengue patients (4–8). Nevertheless, the exact pathophysiology of autoimmune manifestations in patients with dengue infection and the risk of autoimmune diseases are not fully elucidated (9). Previous studies revealed that dengue was associated with several autoimmune disorders, such as Reiter's syndrome, multiple sclerosis, myasthenia gravis, autoimmune encephalomyelitis, systemic vasculitis, systemic lupus erythematosus (SLE), and primary adrenocortical insufficiency (9, 10). Li HM et al. revealed that among patients with dengue infection, the incidence of SARDs was highest for SLE (3.47 cases per 10,000 person-years), followed by rheumatoid arthritis (RA; 2.4 cases per 10,000 person-years), Sjogren's syndrome (SS; 1.07 cases per 10,000 person-years), systemic sclerosis (SSc; 0.27 cases per 10,000 person-years), and inflammatory myopathy (0.27 cases per 10,000 person-years) (10). The authors found that the risk of SLE for patients in the dengue group was significantly higher than that of patients in the non-dengue control group (3). Few studies have investigated the influence of dengue on the development of autoimmune diseases (10, 11). However, to the best of our knowledge, no population-based, case-control study assessed the association between dengue infection and the risk of SARDs. Because the incidences of some SARDs were quite low (for example, polymyositis/dermatomyositis: 0.6–0.7 per 105 person-years) in Taiwan, we are unlikely to identify a sufficient number of patients with a SARD with a very low incidence using a cohort study design (12).

The National Health Insurance Database (NHIRD) has been used for population-based, longitudinal epidemiologic studies over the years in Taiwan. This nationwide, case-control study aimed to determine the association between a history of clinically diagnosed dengue infection and the risk of SARDs.

## Methods

### Ethics

This study was approved by the Institutional Review Board of the Taichung Veterans General Hospital (number CE16251A-2). Patients' personal information was anonymized before data analysis; therefore, informed consent was not required.

### Study Design

This was a nationwide, population-based case-control study based on medical claims data.

### Data Source

Taiwan's National Health Insurance (NHI) program was started in 1995 to ensure the provision of healthcare services for more than 99% of its population. This program collected data on prescription medication, traditional Chinese medical services, catastrophic illness certificates, dental services, ambulatory care, and inpatient services. The Taiwanese National Health Research Institute manages the NHIRD and makes it available for research purposes. All patient's data were anonymized before analyses. The Bureau of the NHI (BNHI) regularly audits the diagnoses recorded *via* reviews of original medical charts, laboratory data, imaging, and pathology reports carried out by at least two independent specialists. The BNHI set up a catastrophic illness registry (CIR) to include patients with major illnesses such as malignancy and autoimmune diseases, which include SLE, RA, SS, DM, PM, and SSc. Two independent specialists first validate the diagnoses after reviewing medical charts and then certify the catastrophic illnesses in the registry. Patients enrolled in the CIR were exempted from copayment.

The NHIRD also constructed a representative database of 1,000,000 individuals, which consisted of individuals randomly selected from all beneficiaries who benefited from services in 2,000, known as the Longitudinal Health Insurance Database (LHID2000).

### Study Samples

**Figure 1:**
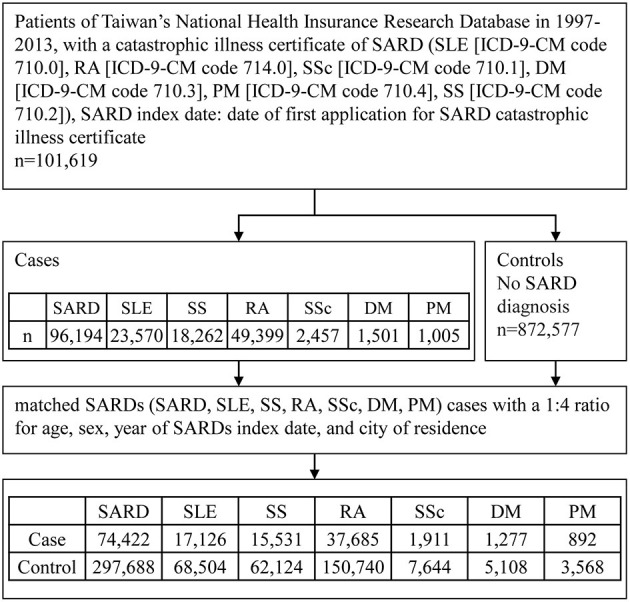
Flowchart of study participant enrollment procedure. SARD, systemic autoimmune rheumatic disease; SLE, systemic lupus erythematosus; RA, rheumatoid arthritis; SSc, systemic sclerosis, scleroderma; DM, dermatomyositis; PM, polymyositis; SS, Sjogren's syndrome; ICD-9-CM, International Classification of Diseases, Ninth Revision, Clinical Modification.

Since this was a retrospective study, the sample size was not calculated. The flowchart of the study subject enrollment is shown in Figure 1. We identified patients with SARDs from the CIR using the 1997–2013 NHIRD. After excluding patients with overlapping diagnoses of SARDs from the CIR, we identified 96,194 patients newly diagnosed with SARDs, including SLE (International Classification of Diseases, Ninth Revision, Clinical Modification [ICD-9-CM] code 710.0), RA (ICD-9-CM code 714.0), SS (ICD-9-CM code 710.2), DM (ICD-9-CM code 710.3), PM (ICD-9-CM code 710.4), and SSc (ICD-9-CM code 710.1), from 1997 to 2013 as the SARD group. From the LHID2000, we identified 872,577 individuals without SARDs. We matched the participants in the SARDs group (SARD, SLE, SS, RA, SSc, DM, PM) with those without SARDs for age, sex, year of SARDs index date, and city of residence in a 1:4 ratio, and finally included 74,422 patients with SARDs and 297,688 non-SARD controls in the study.

### Definition of Dengue Infection

Dengue infection was diagnosed clinically, and it was defined if the patient had one or more outpatient or inpatient visits with a diagnosis of dengue (ICD-9-CM code 061). Given that dengue infection is a notifiable infectious disease in Taiwan, physicians are forced to report cases of dengue to the Taiwanese Centers for Disease Control.

### Potential Confounders

These included the urbanization level of the residence, insured amount according to the payroll, and selected comorbidities within 1 year before the index date. Patients who had at least three ambulatory visits or one inpatient visit with the corresponding ICD-9-CM codes were identified as those with the selected comorbidities (Supplemental Table A). Payroll-related insured amount was used as a proxy measure of an individual's socioeconomic status, and it was divided into quantiles. We used the Deyo et al. revised version of the clinical comorbidity index to represent the general comorbid condition (13).

### Statistical Analyses

We presented continuous variables as mean ± standard deviation and categorical variables as frequencies and percentages. We tested the statistical significance of the associations between two groups using the Student's *t*-test for continuous variables and the Pearson's χ^2^ test for categorical variables. Using the conditional logistic regression analysis, we examined the association between the risk of SARD and a history of dengue infection, which were measured using odds ratios (ORs) with 95% confidence intervals (CIs). We also adjusted potential confounders such as selected comorbidities, urbanization, and income. Subgroup analyses were conducted based on SARD diagnosis. We performed all statistical analyses using SAS version 9.3 (SAS Institute, Inc., Cary, NC, USA). A two-tailed *p* < 0.05 was considered statistically significant.

## Results

### Characteristics of the Study Population

For the total of 74,422 patients newly diagnosed with SARDs in the study group, including 17,126 patients with SLE, 15,531 patients with SS, 37,685 patients with RA, 1,911 patients with SSc, 1,277 patients with DM, and 892 patients with PM, there were 297,688 matched randomly selected controls (non-SARD patients), as shown in Figure 1. Table 1 and Supplementary Tables B–D show their baseline characteristics. The ages of the non-SARD and SARD patients ranged from 21 to 65 years. We found a significantly higher proportion of comorbidities among SARD patients. These comorbidities were hypertension, hyperlipidemia, coronary artery disease, osteoporosis, cerebral vascular accident, asthma, chronic obstructive pulmonary disease, chronic kidney disease, chronic liver disease, hyperthyroidism, thyroiditis, idiopathic thrombocytopenia, autoimmune hemolytic anemia, thrombocytopenia, and antiphospholipid syndrome. The time-lapse between dengue infection and index date of SARD development ranged from 0.2 to 6.6 years.

**Table 1:** Demographic and clinical data of patients with SARDs and controls.

	**Non-SARD**	**SARD**	***P*-value**
***n***	297,688	74,422	
**Gender**			>0.999
Female	234,892 (78.9)	58,723 (78.9)	
Male	62,796 (21.1)	15,699 (21.1)	
SARD age, years	48.3 ± 17.2	48.3 ± 17.2	>0.999
**Dengue history**			<0.001
No	297,438 (99.9)	74,301(99.8)	
Yes	250 (0.1)	121 (0.2)	
**Interval from dengue diagnosis date to the index date, years**			<0.001
Mean ± SD	5.5 ± 3.2	3.9 ± 3.7	
Median (IQR)	5.4 (2.5–8.4)	2.8 (0.2–6.6)	
Range, minimum–maximum	0.1–11.5	0.01–11.3	
**Dengue history group based on interval from dengue diagnosis to the index date**			<0.001
<3 months	5 (0.002)	31 (0.04)	
3 months−1 year	17 (0.01)	12 (0.02)	
1–3 years	43 (0.01)	19 (0.03)	
≥3 years	185 (0.1)	59 (0.1)	
**Urbanization**			0.053
Urban	90,485 (30.4)	22,515 (30.3)	
Suburban	137,734 (46.3)	34,781 (46.7)	
Rural	69,469 (23.3)	17,126 (23.0)	
**Payroll-related insured amount, NTDs**			0.075
Q1 (0)	85,406 (28.7)	21,637 (29.1)	
Q2 (0–19,200)	67,375 (22.6)	16,612 (22.3)	
Q3 (19,200–28,800)	73,078 (24.5)	18,124 (24.4)	
Q4 (>28,800)	71,829 (24.1)	18,049 (24.3)	
**Comorbidity within 1 year before the index date**			
Hypertension	40,808 (13.7)	12,568 (16.9)	<0.001
Diabetes mellitus	19,909 (6.7)	4,758 (6.4)	0.004
Hyperlipidemia	14,380 (4.8)	4,595 (6.2)	<0.001
Coronary artery disease	10,095 (3.4)	3,559 (4.8)	<0.001
Osteoporosis	3,964 (1.3)	3,454 (4.6)	<0.001
Cerebral vascular accident	7,142 (2.4)	2,221 (3)	<0.001
Asthma	3,554 (1.2)	1,919 (2.6)	<0.001
COPD	8,838 (3.0)	4,919 (6.6)	<0.001
Chronic kidney disease	2,187 (0.7)	1,098 (1.5)	<0.001
Chronic liver diseases	7,230 (2.4)	5,662 (7.6)	<0.001
Hyperthyroidism	1,267 (0.4)	899 (1.2)	<0.001
Thyroiditis	156 (0.1)	497 (0.7)	<0.001
ITP	32 (0.01)	618 (0.8)	<0.001
AIHA	3 (0.001)	303 (0.4)	<0.001
Thrombocytopenia	172 (0.1)	1,022 (1.4)	<0.001
Antiphospholipid syndrome	22 (0.01)	73 (0.1)	<0.001

*Results are presented as frequencies (%). SARD, systemic autoimmune rheumatic disease; NTDs, New Taiwan dollars; IQR, interquartile range; Q, quartile; COPD, chronic obstructive pulmonary disease; ITP, idiopathic thrombocytopenia purpura; AIHA, autoimmune hemolytic anemia*.

### Distribution of Months at Dengue Diagnosis

As shown in Supplementary Figures 1–7, the distributions of months at dengue diagnosis were similar between patients with SARD, SLE, SS, RA, SSc, DM, and PM, and the corresponding control groups. The proportion of people diagnosed with dengue among patients with SARDs was highest in October and lowest in March. The proportion of patients diagnosed with dengue among those with SARDs was below 10% from January to June and December. The percentages were around 10–20% from August to November. Taken together, the trend in dengue diagnosis was compatible with the epidemics of reported dengue fever in Taiwan (14).

### Associations Between the Risk of SARD and a History of Dengue Infection

As shown in Tables 2, 3, the associations between the development of SARD and a prior a history of dengue infection were evaluated using conditional logistic regression analysis. The risk of SARD was significantly associated with a history of dengue infection (OR, 1.53; 95% CI, 1.20–1.94; *p* = 0.001), especially in those with SLE (OR, 4.55; 95% CI, 2.77–7.46; *p* < 0.001). No significant association was found between a history of dengue infection and the risk of SS (OR, 1.41; 95% CI, 0.88–2.26; *p* = 0.155), RA (OR, 1.03; 95% CI, 0.70–1.50; *p* = 0.888), SSc (OR, 1.97; 95% CI, 0.38–10.29; *p* = 0.420), DM (OR, 0.54; 95% CI, 0.04–7.27; *p* = 0.641), or PM (OR, 2.08; 95% CI, 0.23–18.79; *p* = 0.513). Associations between other variables and the risk of SLE, SS, RA, SSc, DM, and PM are shown in Supplementary Tables E–G.

**Table 2:** Associations between variables and the risk of SARDs.

	**Univariable**	**Multivariable**	
		**Model 1**	**Model 2**
**Variable**	**OR**	**aOR**	**aOR**
Dengue history	Ref.	Ref.	Ref.
No			
Yes	1.95 (1.57–2.42)	1.53 (1.20–1.94)	–
**Dengue history group based on interval from dengue diagnosis to the index date**			
<3 months	24.82 (9.65–63.84)		18.56 (6.99–49.25)
3 months−1 year	2.83 (1.35–5.94)		0.83 (0.32–2.12)
1–3 years	1.77 (1.03–3.05)		1.49 (0.82–2.73)
≥3 years	1.28 (0.96–1.72)		1.13 (0.82–1.54)
**Urbanization**			
Urban	Ref.	Ref.	Ref.
Suburban	1.02 (0.99–1.04)	1.02 (0.99–1.04)	1.02 (0.99–1.04)
Rural	0.98 (0.95–1.02)	0.99 (0.96–1.02)	0.99 (0.96–1.02)
**Payroll-related insured amount, NTDs**			
Q1 (0)	Ref.	Ref.	Ref.
Q2 (0–19,200)	0.97 (0.95–0.99)	0.96 (0.93–0.98)	0.96 (0.93–0.98)
Q3 (19,200–28,800)	0.97 (0.95–1.00)	1.00 (0.97–1.02)	1.00 (0.97–1.02)
Q4 (<28,800)	0.99 (0.96–1.01)	0.99 (0.97–1.02)	0.99 (0.97–1.02)
**Comorbidity, within 1 year before the index date**			
Hypertension	1.37 (1.33–1.40)	1.21 (1.17–1.24)	1.21 (1.17–1.24)
Diabetes mellitus	0.95 (0.92–0.98)	0.73 (0.71–0.76)	0.73 (0.71–0.76)
Hyperlipidemia	1.32 (1.28–1.37)	1.18 (1.13–1.22)	1.18 (1.13–1.22)
Coronary artery disease	1.47 (1.42–1.54)	1.21 (1.16–1.27)	1.21 (1.16–1.27)
Osteoporosis	3.92 (3.73–4.11)	3.70 (3.52–3.89)	3.70 (3.52–3.90)
Cerebral vascular accident	1.27 (1.21–1.34)	1.07 (1.01–1.13)	1.07 (1.01–1.13)
Asthma	2.21 (2.09–2.34)	0.93 (0.87–1.01)	0.93 (0.87–1.01)
COPD	2.43 (2.34–2.52)	2.25 (2.14–2.36)	2.25 (2.14–2.36)
Chronic kidney disease	2.04 (1.90–2.20)	1.85 (1.71–2.00)	1.85 (1.71–2.00)
Chronic liver diseases	3.39 (3.27–3.52)	3.08 (2.96–3.20)	3.08 (2.96–3.20)
Hyperthyroidism	2.87 (2.63–3.13)	2.42 (2.20–2.65)	2.42 (2.21–2.65)
Thyroiditis	12.84 (10.73–15.38)	11.24 (9.33–13.53)	11.25 (9.34–13.55)
ITP	77.86 (54.57–111.09)	50.50 (34.98–72.89)	50.48 (34.97–72.87)
AIHA	406.28 (130.30–>999)	289.70 (92.64–905.98)	289.34 (92.52–904.88)
Thrombocytopenia	24.16 (20.55–28.41)	16.05 (13.56–19.00)	16.13 (13.63–19.10)
Antiphospholipid syndrome	13.31 (8.26–21.45)	9.41 (5.68–15.57)	9.26 (5.59–15.35)

*Analyses were conducted using the conditional logistic regression model. The matched variables included age, sex, year of SARDs index date, and city of residence. In the multivariable analyses, all variables were adjusted unless specified otherwise*.

*OR, odds ratio; aOR, adjusted odds ratio; CI, confidence interval; SARD, systemic autoimmune rheumatic disease; NTDs, New Taiwan dollars; Q, quartile; IQR, interquartile range; COPD, chronic obstructive pulmonary disease; ITP, idiopathic thrombocytopenia purpura; AIHA,autoimmune hemolytic anemia*.

**Table 3:** Associations between a history of dengue infection and the risk of systemic lupus erythematosus, Sjögren's syndrome, rheumatoid arthritis, systemic sclerosis, dermatomyositis, and polymyositis.

	**SLE**	**SS**	**RA**
	**aOR (95% CI)**	**aOR (95% CI)**	**aOR (95% CI)**
Dengue history	4.55 (2.77–7.46)	1.41 (0.88–2.26)	1.03 (0.70–1.50)
	**SSc**	**DM**	**PM**
	**aOR (95% CI)**	**aOR (95% CI)**	**aOR (95% CI)**
Dengue history	1.97 (0.38–10.29)	0.54 (0.04–7.27)	2.08 (0.23–18.79)

*Analyses were conducted using the conditional logistic regression model. The matched variables included age, sex, year of SARDs index date, and city of residence. Adjusted variables included urbanization, payroll-related insured amount, and selected comorbidities, including hypertension, diabetes mellitus, hyperlipidemia, coronary artery disease, osteoporosis, cerebral vascular accident, asthma, chronic obstructive pulmonary disease, chronic kidney disease, chronic liver disease, hyperthyroidism, thyroiditis, idiopathic thrombocytopenia purpura, autoimmune hemolytic anemia, thrombocytopenia, and antiphospholipid syndrome*.

*OR, odds ratio; aOR, adjusted odds ratio; CI, confidence interval; SLE, systemic lupus erythematosus; SS, Sjogren's syndrome; RA, rheumatoid arthritis; SSc, systemic sclerosis, scleroderma; DM, dermatomyositis; PM, polymyositis*.

### Associations Between the Risk of SARD and a History of Dengue Based on the Time-Lapse From Dengue Diagnosis to the Index Date

As shown in Tables 2, 4, the associations between a history of dengue and the risk of SARD and SS were significant when the time-lapse from dengue diagnosis to the index date was less than 3 months (OR, 18.56; 95% CI, 6.99–49.25 for SARD; OR, 12.97; 95% CI, 1.19–141.02 for SS). However, the risk of SLE was significantly associated with a history of dengue when the time-lapse from dengue diagnosis to the index date was 1–3 years (OR, 4.89; 95% CI, 1.54–15.58).

**Table 4:** Associations between dengue history based on the interval from dengue diagnosis to the index date and the risk of systemic lupus erythematosus, Sjogren's syndrome, rheumatoid arthritis, systemic sclerosis, dermatomyositis, and polymyositis.

	**SLE**	**SS**	**RA**
	**aOR (95% CI)**	**aOR (95% CI)**	**aOR (95% CI)**
**Dengue history group based on interval from dengue diagnosis to the index date**			
Without dengue history	Ref.	Ref.	Ref.
<3 months	NC	12.97 (1.19–141.02)	3.07 (0.60–15.64)
3 months−1 year	NC	0.24 (0.01–4.06)	1.65 (0.66–4.11)
1–3 years	4.89 (1.54–15.58)	0.39 (0.06–2.77)	1.16 (0.42–3.15)
≥3 years	1.60 (0.75–3.41)	1.53 (0.91–2.58)	0.82 (0.50–1.34)
	**SSc**	**DM**	**PM**
	**aOR (95% CI)**	**aOR**	**aOR**
**Dengue history group based on interval from dengue diagnosis to the index date**			
Without Dengue history	Ref.	Ref.	Ref.
<3 months		2.04 (0.04–120.67)	NC
3 months−1 year		NC	
1 year−3 years	2.22 (0.20–24.68)	NC	NC
≥3 years	1.78 (0.18–17.47)	NC	3.03 (0.19–48.68)

*Analyses were conducted using the conditional logistic regression model. The matched variables included age, sex, year of SARDs index date, and city of residence. The adjusted variables were urbanization, payroll-related insured amount, and selected comorbidities, including hypertension, diabetes mellitus, hyperlipidemia, coronary artery disease, osteoporosis, cerebral vascular accident, asthma, chronic obstructive pulmonary disease, chronic kidney disease, chronic liver disease, hyperthyroidism, thyroiditis, idiopathic thrombocytopenia purpura, autoimmune hemolytic anemia, thrombocytopenia, and antiphospholipid syndrome*.

*OR: odds ratio; aOR: adjusted odds ratio; CI: confidence interval; SLE: systemic lupus erythematosus; SS: Sjogren's syndrome; RA: rheumatoid arthritis; SSc: systemic sclerosis, scleroderma; DM: dermatomyositis; PM: polymyositis. NC: not calculable*.

## Discussion

To the best of our knowledge, this is the first nationwide, population-based case-control study to examine the association between a history of dengue infection and the risk of SARD. An essential finding is that there is a significant association between a history of dengue infection and the subsequent development of SARD, especially SLE (OR, 4.55; 95% CI, 2.77–7.46; *p* < 0.001). However, dengue infection a history was not significantly associated with SS, RA, SSc, DM, or PM.

Dengue infection was attributed to multiple immune responses, such as the contribution of autoantibodies against the DENV NS1 antigen, the DENV precursor membrane, and E proteins. Those autoantibodies also interact with self-antigens such as platelets, plasminogen, and integrin (9). Li HM et al. conducted a cohort study that identified an association between dengue infection and the development of autoimmune diseases, including SLE, Reiter's syndrome, multiple sclerosis, myasthenia gravis, autoimmune encephalomyelitis, systemic vasculitis, and primary adrenocortical insufficiency (10). Several case reports revealed that the infection occurred following autoimmune diseases, such as SLE, lupus nephritis, necrotizing scleritis, multifocal motor neuropathy, neuromyelitis optica spectrum disorder, longitudinally extensive transverse myelitis, anti-glomerular basement membrane disease (15–24). Dengue myopathy was also reported (25). We hypothesized that DENV infection triggers autoimmunity. Although the exact association between dengue infection and the development of SARDs remains unclear, several prior studies suggested that dengue infection and SLE may share some pathogenetic mechanisms and genetic backgrounds. DENV NS1-induced autophagy was noted in a study of vascular leakage and autophagy inhibitors (4). Autophagy was associated with immune-related renal diseases, including lupus nephritis (26). DENV may lead to the activation of the complement pathway (5, 27), which, together with its resulting dysregulation of B and T cells (28), is involved in the pathogenesis of SLE. Elevated levels of cytokines such as TNF-α and IFN-γ were noted in patients with dengue infection (6). It is worth noting that SLE patients had increased levels of TNF-α, which may play a role in the development of SLE (7), and the IFN-γ-inducible gene was also found in naïve SLE (8). Regarding shared susceptible genes, the CTLA-4 +49 G allele was found to increase the risk of dengue infection and the viral load (29, 30), and was also indicated to be associated with autoimmune diseases and SLE (31, 32). Anti-NS1 antibodies were associated with dengue-induced apoptosis (33). Apoptosis-derived membrane vesicles were found to drive the cyclic guanosine monophosphate–AMP synthase and the stimulator of interferon genes pathway, which resulted in enhancement of type I IFN production in SLE patients (34). Type I IFN played a more important role in the pathogenesis of SLE than in that of other diseases (35), which could explain the significant association between dengue infection and SLE. There were more cases of SLE (17,126 patients) than of other diseases, including SS (15,531 patients), SSc (1,911 patients), DM (1,277 patients), and PM (892 patients). It is possible that the OR became non-significant after adjustments for too many covariates.

The risk of SLE was significantly associated with a history of dengue when the time-lapse from the onset of dengue infection to the index date was 1–3 years (OR, 4.89; 95% CI, 1.54–15.58). However, the OR was significant for SARD (OR, 18.56; 95% CI 6.99–49.25) and SS (OR, 12.97; 95% CI, 1.19–141.02) when the time-lapse from the moment of dengue diagnosis to the index date was <3 months. Autoimmunity and autoantibodies may be triggered by dengue infection. While chronic symptoms persisted for weeks to months, SARDs may develop before the onset of dengue infection and be diagnosed during the treatment of dengue or follow-up, which means early SARD is a risk factor for dengue infection. Some SS and SARD patients had an insidious onset, which might have led to delayed diagnoses. Therefore, we cannot exclude the possibility of the existence of reverse causality or reciprocal causation in the correlation between dengue infection and SARDs (36). The markedly strong association (OR = 18.56) found when the interval between dengue infection and SARD diagnosis was <3 months but not when the interval was ≥3 months suggested that clinically diagnosed-dengue infection may be a warning rather than a trigger factor for SARD.

Inconsistent with the findings of our study, a recent cohort study conducted by Chang CC et al. reported a decreased risk of primary SS development after dengue infection (11). The authors excluded patients who were diagnosed with autoimmune diseases within1 year before the diagnosis of dengue infection to confirm the diagnosis of primary SS (11). However, given that some primary SS patients may present with other autoimmune diseases, such as Hashimoto's thyroiditis and immune thrombocytopenia purpura, before the diagnosis of SS, the exclusion of patients with other autoimmune diseases before the onset of dengue may lead to an underestimation of the incidence of primary SS in patients with dengue. Also, in Chang's study, possible enrolment of patients with mild dengue infection or patients with SARDs other than primary SS may have underestimated the risk of SS in patients with dengue infection. In contrast, although we excluded patients with overlapping SARDs from the CIR, we cannot exclude the possibility that some patients with SS included in our study actually had secondary SS given that some of them with overlapping SARDs only had one catastrophic illness certificate. Furthermore, in our study, the adjustment for some potential confounders that were not adjusted for in the study conducted by Chang CC et al. including urbanization level of the residence area, payroll-related insured amount, and selected comorbidities, might be another explanation for the inconsistent results.

Although a nationwide, population-based cohort has minimal selection bias, some limitations should be mentioned. First, the NHIRD lacked some personal data, including BMI and alcohol and tobacco consumption, which may be potential confounding factors. Second, the accuracy of the diagnoses according to the claim data is a concern. The incidence of dengue may also have been underestimated due to subtle symptoms. Dengue-related symptoms such as headache, arthralgia, or myalgia may mimic SARD-related presentations. Third, for clinically diagnosed dengue infection, we had limited information on laboratory data for confirmation of dengue diagnosis. However, dengue, chikungunya, and hantavirus are notifiable diseases in Taiwan; when a patient is reported to have a notifiable disease, Taiwanese centers for disease control collect blood for analysis. Anti-dengue antibody (IgM and IgG) tests and real-time reverse transcription PCR assays for DENV were arranged for each patient. Moreover, there was no patient code for dengue occurring concurrently with chikungunya (ICD-9-CM code 066.3), hantavirus (ICD-9-CM code 097.81), or severe fever with thrombocytopenia (ICD-9-CM code 069.5). However, clinical data or laboratory reports cannot be approached in the claim data to determine the accuracy of the diagnosis of dengue or other viral infections. Although misclassification of dengue diagnoses might occur, such non-differential misclassification biases in both groups were always toward the null (37). Therefore, in the present study, the risk of SARDs associated with dengue infection might have been underestimated. In contrast, the diagnosis of SARD raised fewer concerns due to its validation by two independent rheumatologists before a catastrophic illness certificate was issued. It is worth noting that the accuracy of coding improved after regular checks of the original medical records by the BNHI (38).

## Conclusion

This nationwide, population-based case-control study revealed that a history of dengue infection was significantly associated with the risk of SARDs, in particular SLE. The association was markedly strong when the interval between dengue infection and SARD diagnosis was <3 months but turned to be non-significant when the interval was ≥3 months. The results suggested that clinically-diagnosed dengue infection may be more likely a warning rather than a trigger factor for SARD (especially SLE and SS). Further clinical and basic studies are needed to elucidate the association between dengue infection and SARDs.

## Data Availability Statement

The original contributions presented in the study are included in the article/Supplementary Material, further inquiries can be directed to the corresponding author.

## Author Contributions

Y-WC: drafted the manuscript. T-YH and H-HC: revised the manuscript. C-HL, H-MC, and C-CL: contributed materials and analysis tools. All authors contributed to the article and approved the submitted version.

## Conflict of Interest

The authors declare that the research was conducted in the absence of any commercial or financial relationships that could be construed as a potential conflict of interest.

## Publisher's Note

All claims expressed in this article are solely those of the authors and do not necessarily represent those of their affiliated organizations, or those of the publisher, the editors and the reviewers. Any product that may be evaluated in this article, or claim that may be made by its manufacturer, is not guaranteed or endorsed by the publisher.
