# Supplementary material for: Association Between a History of Dengue Fever and the Risk of Systemic Autoimmune Rheumatic Diseases: A Nationwide, Population-Based Case-Control Study
Source: Front Med (Lausanne). 2021 Nov 3;8:738291. doi: 10.3389/fmed.2021.738291 (PMC8597927; doi:10.3389/fmed.2021.738291)
Supplement: Supplementary file 1 [file Data_Sheet_1.docx]

**Association between a history of dengue fever and the risk of the risk of systemic autoimmune rheumatic diseases: a nationwide, population-based, case-control study**

Yun-Wen Chen, Tsu-Yi Hsieh, Ching-Heng Lin, Hsian-Min Chen, Chi-Chien Lin, Hsin-Hua Chen

**Supplemental materials**

**Supplementary table A. Selected comorbidities**

| **Selected comorbidities** | **ICD-9-CM code** |
| --- | --- |
| Hypertension | 401–405 |
| Diabetes mellitus | 250 |
| Hyperlipidemia | 272.0–272.4 |
| Coronary artery disease | 410–414 |
| Osteoporosis | 733 |
| Cerebral vascular accident | 430–438 |
| Asthma | 493 |
| Chronic obstructive pulmonary disease | 490–496 |
| Chronic kidney disease | 585 |
| Chronic liver diseases | 571, 573 |
| Hyperthyroidism | 242 |
| Thyroiditis | 245 |
| Idiopathic thrombocytopenia | 287.3 |
| Autoimmune hemolytic anemia | 283.0 |
| Thrombocytopenia | 287.5 |
| Antiphospholipid syndrome | 289.8 |

**Supplementary table B. Demographic data and clinical among patients with systemic lupus erythematosus, Sjogren's syndrome and their controls**

|  | **None-SLE** | **SLE** | **P value** | **None-SS** | **SS** | **P-value** |
| --- | --- | --- | --- | --- | --- | --- |
| **Variable** | n = 68,504 | n = 17,126 |  | n = 62,124 | n = 15,531 |  |
| **Gender** |  |  | >0.999 |  |  | >0.999 |
| Female | 59,272 (86.5) | 14,818 (86.5) |  | 54,008 (86.9) | 13,502 (86.9) |  |
| Male | 9,232 (13.5) | 2,308 (13.5) |  | 8,116 (13.1) | 2,029 (13.1) |  |
| **SARD age, years** | 35.4±16.2 | 35.4±16.2 | >0.999 | 53.2±14.6 | 53.2±14.6 | >0.999 |
| **Dengue history** |  |  | <0.001 |  |  | 0.015 |
| **No** | 68,469 (99.9) | 17,076 (99.7) |  | 62,053 (99.9) | 15,501 (99.8) |  |
| **Yes** | 35 (0.1) | 50 (0.3) |  | 71 (0.1) | 30 (0.2) |  |
| **Interval from dengue diagnosis date to the index date, years** |  |  | <0.001 |  |  | 0.570 |
| Mean ± SD | 6.3±3.3 | 2.1±2.6 |  | 6.4±3.2 | 6.0±3.6 |  |
| Median (IQR) | 6.2 (3.9–9.4) | 1.0 (0.1–3.7) |  | 7.23 (3.26–9.33) | 5.88 (3.86–9.95) |  |
| Range, minimum–maximum | 1.1–11.5 | 0.02–11.3 |  | 0.24–11.54 | 0.06–10.87 |  |
| **Dengue history group based on interval from dengue diagnosis to the index date** |  |  | <0.001 |  |  | 0.002 |
| <3 months | 0 (0.0) | 22 (0.1) |  | 1 (0.002) | 4 (0.03) |  |
| 3month–1 year | 0 (0.0) | 3 (0.02) |  | 2 (0.003) | 1 (0.01) |  |
| 1 year–3 year | 6 (0.01) | 11 (0.1) |  | 14 (0.02) | 2 (0.01) |  |
| ≥3 year | 29 (0.04) | 14 (0.1) |  | 54 (0.1) | 23 (0.1) |  |
| **Urbanization** |  |  | 0.304 |  |  | <0.001 |
| Urban | 21,397 (31.2) | 5,247 (30.6) |  | 19,344 (31.1) | 4,917 (31.7) |  |
| Suburban | 32,443 (47.4) | 8,163 (47.7) |  | 28,224 (45.4) | 7,307 (47) |  |
| Rural | 14,664 (21.4) | 3,716 (21.7) |  | 14,556 (23.4) | 3,307 (21.3) |  |
| **Payroll-related insured amount, NTDs** |  |  | <0.001 |  |  | <0.001 |
| Q1 (0) | 23,456 (34.2) | 5,920 (34.6) |  | 16,488 (26.5) | 4,073 (26.2) |  |
| Q2 (0-19200) | 11,147 (16.3) | 3,001 (17.5) |  | 14,938 (24.0) | 3,399 (21.9) |  |
| Q3 (19200-28800) | 16,736 (24.4) | 4,148 (24.2) |  | 16,066 (25.9) | 3,749 (24.1) |  |
| Q4 (<28800) | 17,165 (25.1) | 4,057 (23.7) |  | 14,632 (23.6) | 4,310 (27.8) |  |
| **Comorbidity, within one year before the index date** |  |  |  |  |  |  |
| Hypertension | 3,582 (5.2) | 1,904 (11.1) | <0.001 | 11,575 (18.6) | 3,120 (20.1) | <0.001 |
| Diabetes mellitus | 1,713 (2.5) | 469 (2.7) | 0.077 | 5,525 (8.9) | 1,004 (6.5) | <0.001 |
| Hyperlipidemia | 1,303 (1.9) | 631 (3.7) | <0.001 | 4,360 (7.0) | 1,378 (8.9) | <0.001 |
| Coronary artery disease | 869 (1.3) | 474 (2.8) | <0.001 | 2,694 (4.3) | 1,019 (6.6) | <0.001 |
| Osteoporosis | 371 (0.5) | 294 (1.7) | <0.001 | 1,088 (1.8) | 766 (4.9) | <0.001 |
| Cerebral vascular accident | 608 (0.9) | 500 (2.9) | <0.001 | 1,826 (2.9) | 629 (4) | <0.001 |
| Asthma | 479 (0.7) | 278 (1.6) | <0.001 | 879 (1.4) | 430 (2.8) | <0.001 |
| COPD | 1,050 (1.5) | 741 (4.3) | <0.001 | 2,202 (3.5) | 1,298 (8.4) | <0.001 |
| Chronic kidney disease | 216 (0.3) | 427 (2.5) | <0.001 | 570 (0.9) | 197 (1.3) | <0.001 |
| Chronic liver diseases | 806 (1.2) | 1,298 (7.6) | <0.001 | 1,746 (2.8) | 1,482 (9.5) | <0.001 |
| Hyperthyroidism | 235 (0.3) | 249 (1.5) | <0.001 | 334 (0.5) | 282 (1.8) | <0.001 |
| Thyroiditis | 25 (0.04) | 157 (0.9) | <0.001 | 47 (0.1) | 210 (1.4) | <0.001 |
| ITP | 3 (0.004) | 497 (2.9) | <0.001 | 9 (0.01) | 92 (0.6) | <0.001 |
| AIHA | 1 (0.001) | 282 (1.6) | <0.001 | 0 (0.0) | 14 (0.1) | <0.001 |
| Thrombocytopenia | 16 (0.02) | 805 (4.7) | <0.001 | 43 (0.1) | 145 (0.9) | <0.001 |
| Antiphospholipid syndrome | 2 (0.003) | 31 (0.2) | <0.001 | 10 (0.02) | 28 (0.2) | <0.001 |

Results are shown as numbers (%). Abbreviations: SLE: systemic lupus erythematosus; SS: Sjogren’s syndrome; SARD: systemic autoimmune rheumatic diseases; NTDs: New Taiwan dollars; Q, quartile; IQR, interquartile range; COPD: chronic obstructive pulmonary disease; ITP: idiopathic thrombocytopenia; AIHA: autoimmune hemolytic anemia

**Supplementary table C. Demographic and clinical data among patients with rheumatoid arthritis and systemic sclerosis, and their controls**

|  | **None-RA** | **RA** | **P-value** | **None-SSc** | **SSc** | **P-value** |
| --- | --- | --- | --- | --- | --- | --- |
| **Variable** | n = 150,740 | n = 37,685 |  | n = 7,644 | n = 1,911 |  |
| **Gender** |  |  | >0.999 |  |  | >0.999 |
| Female | 110,736 (73.5) | 27,684 (73.5) |  | 5,384 (70.4) | 1,346 (70.4) |  |
| Male | 40,004 (26.5) | 10,001 (26.5) |  | 2,260 (29.6) | 565 (29.6) |  |
| **SARD age, years** | 52.2±15.7 | 52.2±15.7 | >0.999 | 50.9±15.6 | 50.9±15.6 | >0.999 |
| **Dengue history** |  |  | 0.559 |  |  | 0.868 |
| **No** | 150,611 (99.9) | 37,649 (99.9) |  | 7,637 (99.9) | 1,909 (99.9) |  |
| **Yes** | 129 (0.1) | 36 (0.1) |  | 7 (0.1) | 2 (0.1) |  |
| **Dengue diagnosis date to index date, years** |  |  | 0.710 |  |  | 0.779 |
| Mean ± SD | 5.0±3.1 | 4.7±4.0 |  | 5.0±3.1 | 5.8±4.5 |  |
| Median (IQR) | 5.08 (2.28–7.41) | 3.49 (0.91–8.79) |  | 4.57 (2.19–8.44) | 5.77 (2.56–8.98) |  |
| Range, minimum–maximum | (0.10–10.62) | (0.01–10.76) |  | (1.21–9.62) | (2.56–8.98) |  |
| **Dengue history group based on interval from dengue diagnosis to the index date** |  |  | 0.114 |  |  | 0.829 |
| <3 months | 3 (0.002) | 3 (0.01) |  | 0 (0.0) | 0 (0.0) |  |
| 3month–1 year | 14 (0.01) | 8 (0.02) |  | 0 (0.0) | 0 (0.0) |  |
| 1 year–3 year | 19 (0.01) | 5 (0.01) |  | 2 (0.03) | 1 (0.1) |  |
| ≥3 year | 93 (0.1) | 20 (0.1) |  | 5 (0.1) | 1 (0.1) |  |
| **Urbanization** |  |  | 0.673 |  |  | 0.742 |
| Urban | 44,846 (29.8) | 11,131 (29.5) |  | 2,163 (28.3) | 544 (28.5) |  |
| Suburban | 69,406 (46.0) | 17,436 (46.3) |  | 3,636 (47.6) | 892 (46.7) |  |
| Rural | 36,488 (24.2) | 9,118 (24.2) |  | 1,845 (24.1) | 475 (24.9) |  |
| **Payroll-related insured amount, NTDs** |  |  | <0.001 |  |  | 0.947 |
| Q1 (0) | 40,977 (27.2) | 10,509 (27.9) |  | 2,010 (26.3) | 512 (26.8) |  |
| Q2 (0-19200) | 34,550 (22.9) | 8,368 (22.2) |  | 1,863 (24.4) | 470 (24.6) |  |
| Q3 (19200-28800) | 38,430 (25.5) | 9,937 (26.4) |  | 2,013 (26.3) | 499 (26.1) |  |
| Q4 (<28800) | 36,783 (24.4) | 8,871 (23.5) |  | 1,758 (23.0) | 430 (22.5) |  |
| **Comorbidity, a year prior to the index date** |  |  |  |  |  |  |
| Hypertension | 23,415 (15.5) | 6,795 (18) | <0.001 | 1,112 (14.5) | 342 (17.9) | <0.001 |
| Diabetes mellitus | 11,569 (7.7) | 2,957 (7.8) | 0.263 | 555 (7.3) | 142 (7.4) | 0.798 |
| Hyperlipidemia | 7,907 (5.2) | 2,279 (6.0) | <0.001 | 415 (5.4) | 123 (6.4) | 0.088 |
| Coronary artery disease | 5,962 (4.0) | 1,814 (4.8) | <0.001 | 284 (3.7) | 109 (5.7) | <0.001 |
| Osteoporosis | 2,289 (1.5) | 2,261 (6.0) | <0.001 | 100 (1.3) | 61 (3.2) | <0.001 |
| Cerebral vascular accident | 4,319 (2.9) | 972 (2.6) | 0.003 | 200 (2.6) | 58 (3.0) | 0.313 |
| Asthma | 1,998 (1.3) | 1,071 (2.8) | <0.001 | 86 (1.1) | 58 (3.0) | <0.001 |
| COPD | 5,089 (3.4) | 2,456 (6.5) | <0.001 | 244 (3.2) | 183 (9.6) | <0.001 |
| Chronic kidney disease | 1,269 (0.8) | 396 (1.1) | <0.001 | 53 (0.7) | 47 (2.5) | <0.001 |
| Chronic liver diseases | 4,236 (2.8) | 2,254 (6.0) | <0.001 | 222 (2.9) | 167 (8.7) | <0.001 |
| Hyperthyroidism | 641 (0.4) | 302 (0.8) | <0.001 | 34 (0.4) | 25 (1.3) | <0.001 |
| Thyroiditis | 77 (0.1) | 106 (0.3) | <0.001 | 3 (0.04) | 5 (0.3) | 0.003 |
| ITP | 17 (0.01) | 19 (0.1) | <0.001 | 2 (0.03) | 6 (0.3) | <0.001 |
| AIHA | 1 (0.001) | 6 (0.02) | <0.001 | 0 (0.0) | 0 (0.0) | NA |
| Thrombocytopenia | 104 (0.1) | 51 (0.1) | <0.001 | 4 (0.1) | 5 (0.3) | 0.008 |
| Antiphospholipid syndrome | 8 (0.01) | 14 (0.04) | <0.001 | 0 (0.0) | 0 (0.0) | NA |

Results are shown as numbers (%). Abbreviations: RA: rheumatoid arthritis; SSc: systemic sclerosis, scleroderma; SARD: systemic autoimmune rheumatic diseases; NTDs: New Taiwan dollars; Q, quartile; IQR, interquartile range; COPD: chronic obstructive pulmonary disease; ITP: idiopathic thrombocytopenia; AIHA: autoimmune hemolytic anemia; NA: not available

**Supplementary table D. Demographic data among patients of dermatomyositis and polymyositis with or without history of dengue infection**

|  | **None-DM** | **DM** | **P-value** | **None-PM** | **PM** | **P-value** |
| --- | --- | --- | --- | --- | --- | --- |
| **Variable** | n = 5,108 | n = 1,277 |  | n = 3,568 | n = 892 |  |
| **Gender** |  |  | >0.999 |  |  | >0.999 |
| Female | 3,216 (63.0) | 804 (63.0) |  | 2,276 (63.8) | 569 (63.8) |  |
| Male | 1,892 (37.0) | 473 (37.0) |  | 1,292 (36.2) | 323 (36.2) |  |
| **SARD age, years** | 45.2±18.7 | 45.2±18.7 | >0.999 | 49.9±15.8 | 49.9±15.8 | >0.999 |
| **Dengue history** |  |  | 0.705 |  |  | 0.133 |
| **No** | 5,102 (99.9) | 1,276 (99.9) |  | 3,566 (99.9) | 890 (99.8) |  |
| **Yes** | 6 (0.1) | 1 (0.1) |  | 2 (0.1) | 2 (0.2) |  |
| **Interval from dengue diagnosis to the index date, years** |  |  | NA |  |  | 0.777 |
| Mean ± SD | 2.9±2.4 | 0.2±NA |  | 4.0±2.1 | 3.0±3.9 |  |
| Median (IQR) | 2.86 (0.59–4.76) | 0.18 (0.18–0.18) |  | 3.97 (2.52–5.43) | 2.96 (0.18–5.74) |  |
| Range, minimum–maximum | (0.17–6.31) | (0.18–0.18) |  | (2.52–5.43) | (0.18–5.74) |  |
| **Dengue history group based on. time interval from dengue diagnosis to the index date** |  |  | 0.667 |  |  | 0.146 |
| <3 months | 1 (0.02) | 1 (0.1) |  | 0 (0.0) | 1 (0.1) |  |
| 3month-1 year | 1 (0.02) | 0 (0.0) |  | 0 (0.0) | 0 (0.0) |  |
| 1 year–3 year | 1 (0.02) | 0 (0.0) |  | 1 (0.03) | 0 (0.0) |  |
| ≥3 year | 3 (0.1) | 0 (0.0) |  | 1 (0.03) | 1 (0.1) |  |
| **Urbanization** |  |  | 0.167 |  |  | 0.812 |
| Urban | 1,652 (32.3) | 396 (31.0) |  | 1,083 (30.4) | 280 (31.4) |  |
| Suburban | 2,364 (46.3) | 577 (45.2) |  | 1,661 (46.6) | 406 (45.5) |  |
| Rural | 1,092 (21.4) | 304 (23.8) |  | 824 (23.1) | 206 (23.1) |  |
| **Payroll-related insured amount, NTDs** |  |  | 0.218 |  |  | 0.055 |
| Q1 (0) | 1,551 (30.4) | 394 (30.9) |  | 924 (25.9) | 229 (25.7) |  |
| Q2 (0–19200) | 1,142 (22.4) | 311 (24.4) |  | 930 (26.1) | 267 (29.9) |  |
| Q3 (19200–28800) | 1,245 (24.4) | 280 (21.9) |  | 850 (23.8) | 182 (20.4) |  |
| Q4 (<28800) | 1,170 (22.9) | 292 (22.9) |  | 864 (24.2) | 214 (24.0) |  |
| **Comorbidity within one year before the index date** |  |  |  |  |  |  |
| Hypertension | 605 (11.8) | 213 (16.7) | <0.001 | 519 (14.5) | 194 (21.7) | <0.001 |
| Diabetes mellitus | 285 (5.6) | 103 (8.1) | <0.001 | 262 (7.3) | 83 (9.3) | 0.050 |
| Hyperlipidemia | 229 (4.5) | 87 (6.8) | <0.001 | 166 (4.7) | 97 (10.9) | <0.001 |
| Coronary artery disease | 148 (2.9) | 51 (4.0) | 0.044 | 138 (3.9) | 92 (10.3) | <0.001 |
| Osteoporosis | 62 (1.2) | 35 (2.7) | <0.001 | 54 (1.5) | 37 (4.1) | <0.001 |
| Cerebral vascular accident | 101 (2.0) | 33 (2.6) | 0.176 | 88 (2.5) | 29 (3.3) | 0.190 |
| Asthma | 67 (1.3) | 45 (3.5) | <0.001 | 45 (1.3) | 37 (4.1) | <0.001 |
| COPD | 148 (2.9) | 117 (9.2) | <0.001 | 105 (2.9) | 124 (13.9) | <0.001 |
| Chronic kidney disease | 39 (0.8) | 12 (0.9) | 0.527 | 40 (1.1) | 19 (2.1) | 0.018 |
| Chronic liver diseases | 118 (2.3) | 224 (17.5) | <0.001 | 102 (2.9) | 237 (26.6) | <0.001 |
| Hyperthyroidism | 11 (0.2) | 22 (1.7) | <0.001 | 12 (0.3) | 19 (2.1) | <0.001 |
| Thyroiditis | 2 (0.04) | 8 (0.6) | <0.001 | 2 (0.1) | 11 (1.2) | <0.001 |
| ITP | 1 (0.02) | 1 (0.1) | 0.289 | 0 (0.0) | 3 (0.3) | <0.001 |
| AIHA | 1 (0.02) | 0 (0.0) | 0.617 | 0 (0.0) | 1 (0.1) | 0.045 |
| Thrombocytopenia | 3 (0.1) | 10 (0.8) | <0.001 | 2 (0.1) | 6 (0.7) | <0.001 |
| Antiphospholipid syndrome | 1 (0.02) | 0 (0.0) | 0.617 | 1 (0.03) | 0 (0.0) | 0.617 |

Results are shown as numbers (%). Abbreviations: DM: dermatomyositis; PM: polymyositis; SARD: systemic autoimmune rheumatic diseases; NTDs: New Taiwan dollars; Q, quartile; IQR, interquartile range; COPD: chronic obstructive pulmonary disease; ITP: idiopathic thrombocytopenia; AIHA: autoimmune hemolytic anemia; NA: not available. Only 1 patient has a history of dengue infection in patients of dermatomyositis.

**Supplementary table E. Associations between variables and the risk of systemic lupus erythematosus and Sjogren's syndrome**

|  | **SLE** | | | **SS** | | |
| --- | --- | --- | --- | --- | --- | --- |
|  | **Univariable** | **Multivariable** | | **Univariable** | **Multivariable** | |
|  |  | **Model 1** | **Model 2** |  | **Model 1** | **Model 2** |
|  | **OR (95% CI)** | **aOR (95% CI)** | **aOR (95% CI)** | **OR** | **aOR (95% CI)** | **aOR (95% CI)** |
| **Dengue history** |  |  |  |  |  |  |
| **No** | Ref. | Ref. | Ref. | Ref. | Ref. | Ref. |
| **Yes** | 5.71 (3.71–8.80) | 4.55 (2.77–7.46) |  | 1.70 (1.11–2.60) | 1.41 (0.88–2.26) |  |
| **Dengue history group based on interval from dengue diagnosis to the index date** |  |  |  |  |  |  |
| <3 months | NC |  | NC | 16.00 (1.79–143.15) |  | 12.97 (1.19–141.02) |
| 3month–1 year | NC |  | NC | 2.00 (0.18–22.06) |  | 0.24 (0.01–4.06) |
| 1 year–3 years | 7.33 (2.71–19.83) |  | 4.89 (1.54–15.58) | 0.57 (0.13–2.53) |  | 0.39 (0.06–2.77) |
| ≥3 years | 1.93 (1.02–3.65) |  | 1.60 (0.75–3.41) | 1.71 (1.05–2.79) |  | 1.53 (0.91–2.58) |
| **Urbanization** |  |  |  |  |  |  |
| Urban | Ref. | Ref. | Ref. | Ref. | Ref. | Ref. |
| Suburban | 1.05 (0.99–1.10) | 1.05 (0.99–1.10) | 1.05 (0.99–1.10) | 0.98 (0.94–1.03) | 0.99 (0.95–1.05) | 0.99 (0.94–1.05) |
| Rural | 1.07 (0.99–1.14) | 1.03 (0.96–1.11) | 1.03 (0.96–1.11) | 0.81 (0.75–0.86) | 0.83 (0.78–0.90) | 0.83 (0.78–0.90) |
| **Payroll-related insured amount, NTDs** |  |  |  |  |  |  |
| Q1 (0) | Ref. | Ref. | Ref. | Ref. | Ref. | Ref. |
| Q2 (0–19200) | 1.05 (0.99–1.11) | 1.07 (1.01–1.14) | 1.07 (1.01–1.13) | 0.93 (0.88–0.98) | 0.92 (0.87–0.97) | 0.92 (0.87–0.97) |
| Q3 (19200–28800) | 0.96 (0.91–1.02) | 0.98 (0.93–1.04) | 0.98 (0.93–1.04) | 0.95 (0.90–1.01) | 0.98 (0.92–1.04) | 0.98 (0.92–1.04) |
| Q4 (<28800) | 0.91 (0.86–0.96) | 0.94 (0.89–1.003) | 0.94 (0.89–1.002) | 1.24 (1.18–1.31) | 1.23 (1.17–1.31) | 1.24 (1.17–1.31) |
| **Comorbidity within one year before the index date** |  |  |  |  |  |  |
| Hypertension | 3.07 (2.86–3.30) | 2.43 (2.23–2.64) | 2.42 (2.22–2.64) | 1.12 (1.07–1.18) | 0.99 (0.94–1.05) | 0.99 (0.94–1.05) |
| Diabetes mellitus | 1.11 (1.00–1.24) | 0.52 (0.46–0.60) | 0.52 (0.46–0.60) | 0.69 (0.64–0.74) | 0.54 (0.49–0.58) | 0.54 (0.49–0.58) |
| Hyperlipidemia | 2.09 (1.89–2.32) | 1.49 (1.32–1.70) | 1.50 (1.32–1.70) | 1.31 (1.23–1.40) | 1.28 (1.19–1.38) | 1.28 (1.19–1.38) |
| Coronary artery disease | 2.44 (2.16–2.76) | 1.53 (1.32–1.77) | 1.53 (1.32–1.77) | 1.61 (1.49–1.74) | 1.47 (1.35–1.60) | 1.47 (1.35–1.61) |
| Osteoporosis | 3.44 (2.93–4.03) | 3.20 (2.65–3.87) | 3.20 (2.65–3.87) | 3.10 (2.81–3.41) | 2.83 (2.55–3.15) | 2.83 (2.55–3.15) |
| Cerebral vascular accident | 3.78 (3.32–4.29) | 2.82 (2.41–3.30) | 2.82 (2.41–3.29) | 1.43 (1.30–1.57) | 1.31 (1.18–1.45) | 1.31 (1.18–1.45) |
| Asthma | 2.38 (2.05–2.76) | 0.77 (0.62–0.97) | 0.78 (0.62–0.97) | 1.99 (1.77–2.24) | 0.70 (0.60–0.82) | 0.70 (0.60–0.82) |
| COPD | 3.11 (2.82–3.44) | 2.85 (2.45–3.32) | 2.85 (2.45–3.31) | 2.61 (2.43–2.81) | 2.75 (2.50–3.03) | 2.75 (2.50–3.03) |
| Chronic kidney disease | 8.31 (7.03–9.82) | 7.09 (5.86–8.58) | 7.09 (5.86–8.58) | 1.39 (1.18–1.64) | 1.39 (1.16–1.65) | 1.39 (1.16–1.65) |
| Chronic liver diseases | 7.50 (6.83–8.24) | 7.01 (6.31–7.78) | 7.00 (6.30–7.78) | 3.75 (3.48–4.03) | 3.60 (3.33–3.90) | 3.60 (3.33–3.89) |
| Hyperthyroidism | 4.31 (3.60–5.16) | 3.56 (2.89–4.37) | 3.56 (2.90–4.37) | 3.43 (2.92–4.02) | 3.09 (2.60–3.66) | 3.09 (2.60–3.66) |
| Thyroiditis | 25.36 (16.62–38.69) | 23.81 (15.16–37.41) | 23.85 (15.18–37.47) | 17.97 (13.09–24.65) | 16.01 (11.56–22.18) | 16.02 (11.56–22.18) |
| ITP | 671.68 (215.90–>999) | 638.80 (158.64–>999) | 639.67 (158.86–>999) | 41.22 (20.78–81.78) | 30.57 (14.92–62.64) | 31.13 (15.07–64.30) |
| AIHA | NC | 893.78 (122.15–>999) | 893.20 (122.07–>999) | NC | NC | NC |
| Thrombocytopenia | 221.00 (132.57–368.43) | 159.18 (94.82–267.21) | 159.69 (95.13–268.06) | 13.67 (9.72–19.23) | 9.56 (6.57–13.91) | 9.71 (6.66–14.15) |
| Antiphospholipid syndrome | 62.00 (14.84–259.05) | 46.75 (10.78–202.62) | 46.69 (10.77–202.43) | 11.25 (5.46–23.18) | 8.18 (3.79–17.64) | 7.84 (3.62–17.00) |

Abbreviations: OR: odds ratio; aOR: adjusted odds ratios; CI: confidence intervals; NTDs: New Taiwan dollars; Q, quartile; IQR, interquartile range; SLE: systemic lupus erythematosus; SS: Sjogren’s syndrome; NC, not calculable.

**Supplementary table F. Associations between variables and the risk of rheumatoid arthritis and systemic sclerosis**

|  | **RA** | | | **SSc** | | |
| --- | --- | --- | --- | --- | --- | --- |
|  | **Univariable** | **Multivariable** | | **Univariable** | **Multivariable** | |
|  |  | **Model 1** | **Model 2** |  | **Model 1** | **Model 2** |
|  | **OR (95% CI)** | **aOR (95% CI)** | **aOR (95% CI)** | **OR (95% CI)** | **aOR (95% CI)** | **aOR (95% CI)** |
| **Dengue history** |  |  |  |  |  |  |
| **No** | Ref. | Ref. | Ref. | Ref. | Ref | Ref |
| **Yes** | 1.12 (0.77–1.62) | 1.03 (0.70–1.50) |  | 1.14 (0.24–5.50) | 1.97 (0.38–10.29) |  |
| **Dengue history group based on interval from dengue infection to the index date** |  |  |  |  |  |  |
| <3 months | 3.99 (0.81–19.77) |  | 3.07 (0.60–15.64) | NA |  | NA |
| 3month–1 year | 2.29 (0.96–5.48) |  | 1.65 (0.66–4.11) | NA |  | NA |
| 1 year–3 years | 1.05 (0.39–2.83) |  | 1.16 (0.42–3.15) | 2.00 (0.18–22.06) |  | 2.22 (0.20–24.68) |
| ≥3 years | 0.86 (0.53–1.40) |  | 0.82 (0.50–1.34) | 0.80 (0.09–6.85) |  | 1.78 (0.18–17.47) |
| **Urbanization** |  |  |  |  |  |  |
| Urban | Ref. | Ref. | Ref. | Ref. | Ref. | Ref. |
| Suburban | 1.02 (0.99–1.05) | 1.02 (0.99–1.06) | 1.02 (0.99–1.06) | 0.98 (0.84–1.13) | 1.00 (0.85–1.18) | 1.00 (0.85–1.18) |
| Rural | 1.01 (0.97–1.06) | 1.01 (0.97–1.06) | 1.01 (0.97–1.06) | 1.05 (0.86–1.27) | 1.06 (0.85–1.32) | 1.06 (0.85–1.32) |
| **Payroll-related insured amount, NTDs** |  |  |  |  |  |  |
| Q1 (0) | Ref. | Ref. | Ref. | Ref. | Ref. | Ref. |
| Q2 (0-19200) | 0.94 (0.90–0.97) | 0.92 (0.89–0.95) | 0.92 (0.89–0.95) | 0.98 (0.85–1.14) | 0.98 (0.83–1.17) | 0.98 (0.83–1.17) |
| Q3 (19200-28800) | 1.00 (0.97–1.04) | 1.01 (0.98–1.05) | 1.01 (0.98–1.05) | 0.97 (0.83–1.12) | 1.01 (0.85–1.20) | 1.01 (0.85–1.20) |
| Q4 (<28800) | 0.92 (0.89–0.96) | 0.92 (0.89–0.96) | 0.92 (0.89–0.96) | 0.95 (0.81–1.12) | 0.94 (0.78–1.14) | 0.95 (0.78–1.14) |
| **Comorbidity within one year before the index date** |  |  |  |  |  |  |
| Hypertension | 1.25 (1.21–1.29) | 1.16 (1.12–1.21) | 1.16 (1.12–1.21) | 1.35 (1.16–1.56) | 1.12 (0.93–1.34) | 1.12 (0.93–1.35) |
| Diabetes mellitus | 1.03 (0.98–1.07) | 0.88 (0.84–0.92) | 0.88 (0.84–0.93) | 1.03 (0.84–1.25) | 0.95 (0.75–1.21) | 0.95 (0.75–1.21) |
| Hyperlipidemia | 1.17 (1.12–1.23) | 1.08 (1.03–1.14) | 1.08 (1.03–1.14) | 1.21 (0.98–1.50) | 0.98 (0.76–1.27) | 0.98 (0.75–1.27) |
| Coronary artery disease | 1.25 (1.18–1.32) | 1.07 (1.01–1.14) | 1.07 (1.01–1.14) | 1.63 (1.29–2.07) | 1.50 (1.13–2.00) | 1.50 (1.13–2.00) |
| Osteoporosis | 4.51 (4.24–4.79) | 4.30 (4.04–4.58) | 4.30 (4.04–4.58) | 2.55 (1.84–3.55) | 2.73 (1.88–3.95) | 2.72 (1.88–3.95) |
| Cerebral vascular accident | 0.89 (0.83–0.96) | 0.79 (0.73–0.85) | 0.79 (0.73–0.85) | 1.18 (0.87–1.59) | 1.05 (0.73–1.51) | 1.05 (0.73–1.51) |
| Asthma | 2.20 (2.04–2.38) | 1.17 (1.06–1.29) | 1.17 (1.06–1.29) | 2.76 (1.97–3.86) | 0.69 (0.43–1.09) | 0.69 (0.43–1.09) |
| COPD | 2.07 (1.97–2.18) | 1.82 (1.70–1.95) | 1.82 (1.70–1.95) | 3.39 (2.76–4.17) | 3.49 (2.65–4.60) | 3.49 (2.65–4.60) |
| Chronic kidney disease | 1.25 (1.12–1.41) | 1.16 (1.03–1.31) | 1.16 (1.03–1.31) | 3.62 (2.43–5.38) | 3.46 (2.17–5.50) | 3.46 (2.17–5.50) |
| Chronic liver diseases | 2.23 (2.12–2.35) | 2.11 (1.99–2.22) | 2.10 (1.99–2.22) | 3.30 (2.67–4.08) | 2.97 (2.33–3.78) | 2.97 (2.33–3.78) |
| Hyperthyroidism | 1.90 (1.65–2.18) | 1.66 (1.44–1.92) | 1.66 (1.44–1.92) | 2.94 (1.76–4.93) | 2.73 (1.49–5.02) | 2.73 (1.49–5.02) |
| Thyroiditis | 5.52 (4.12–7.41) | 4.90 (3.63–6.62) | 4.90 (3.63–6.62) | 6.67 (1.59–27.90) | 5.17 (1.12–23.90) | 5.17 (1.12–23.90) |
| ITP | 4.47 (2.32–8.60) | 3.43 (1.72–6.85) | 3.44 (1.72–6.86) | 12.00 (2.42–59.45) | 9.27 (1.64–52.36) | 9.27 (1.64–52.35) |
| AIHA | 24.00 (2.89–199.35) | 20.06 (2.37–169.47) | 20.06 (2.37–169.47) | NA |  |  |
| Thrombocytopenia | 1.96 (1.40–2.74) | 1.22 (0.86–1.74) | 1.21 (0.85–1.72) | 5.00 (1.34–18.62) | 1.53 (0.33–7.15) | 1.53 (0.33–7.15) |
| Antiphospholipid syndrome | 7.00 (2.94–16.69) | 6.05 (2.43–15.03) | 6.05 (2.43–15.03) | NA |  |  |

Abbreviations: OR: odds ratio; aOR: adjusted odds ratios; CI: confidence intervals; NTDs: New Taiwan dollars; Q, quartile; IQR, interquartile range; RA: rheumatoid arthritis; SSc: systemic sclerosis, scleroderma; NA: not available

**Supplementary table G. Associations between variables and the risk of dermatomyositis and polymyositis**

|  | **DM** | | | **PM** | | |
| --- | --- | --- | --- | --- | --- | --- |
|  | **Univariable** | **Multivariable** | | **Univariable** | **Multivariable** | |
|  |  | **Model 1** | **Model 2** |  | **Model 1** | **Model 2** |
|  | **OR** | **aOR** | **aOR** | **OR** | **aOR** | **aOR** |
| **Dengue history** |  |  |  |  |  |  |
| **No** | Ref. | Ref. | Ref. | Ref. | Ref. | Ref. |
| **Yes** | 0.67 (0.08–5.54) | 0.54 (0.04–7.27) |  | 4.00 (0.56–28.40) | 2.08 (0.23–18.79) |  |
| **Dengue history group based on interval from dengue infection to the index date** |  |  |  |  |  |  |
| <3 months | 4.00 (0.25–63.95) |  | 2.04 (0.04–120.67) | NC |  | NC |
| 3month–1 year | NC |  | NC | NA |  |  |
| 1 year–3 year | NC |  | NC | NC |  | NC |
| ≥3 year | NC |  | NC | 4.00 (0.25–63.95) |  | 3.03 (0.19–48.68) |
| **Urbanization** |  |  |  |  |  |  |
| Urban | Ref. | Ref. | Ref. | Ref. | Ref. | Ref. |
| Suburban | 1.08 (0.91–1.28) | 1.01 (0.84–1.21) | 1.01 (0.84–1.21) | 0.92 (0.75–1.13) | 0.85 (0.67–1.08) | 0.85 (0.67–1.08) |
| Rural | 1.33 (1.05–1.69) | 1.21 (0.94–1.56) | 1.21 (0.94–1.56) | 0.93 (0.70–1.24) | 0.89 (0.64–1.25) | 0.89 (0.64–1.24) |
| **Payroll-related insured amount, NTDs** |  |  |  |  |  |  |
| Q1 (0) | Ref. | Ref. | Ref. | Ref. | Ref. | Ref. |
| Q2 (0-19200) | 1.06 (0.87–1.29) | 1.08 (0.87–1.33) | 1.08 (0.87–1.33) | 1.16 (0.94–1.45) | 1.13 (0.88–1.46) | 1.13 (0.87–1.45) |
| Q3 (19200-28800) | 0.86 (0.70–1.05) | 0.83 (0.66–1.03) | 0.83 (0.66–1.03) | 0.85 (0.67–1.08) | 0.85 (0.64–1.12) | 0.85 (0.64–1.12) |
| Q4 (<28800) | 0.96 (0.78–1.18) | 1.01 (0.80–1.26) | 1.01 (0.80–1.26) | 0.99 (0.78–1.26) | 0.89 (0.67–1.18) | 0.89 (0.67–1.18) |
| **Comorbidity within one year before the index date** |  |  |  |  |  |  |
| Hypertension | 1.65 (1.36–1.99) | 1.31 (1.04–1.65) | 1.30 (1.03–1.64) | 1.84 (1.50–2.27) | 1.56 (1.19–2.05) | 1.56 (1.19–2.05) |
| Diabetes mellitus | 1.54 (1.21–1.97) | 1.09 (0.81–1.47) | 1.10 (0.82–1.47) | 1.33 (1.01–1.74) | 0.77 (0.54–1.10) | 0.77 (0.54–1.10) |
| Hyperlipidemia | 1.64 (1.25–2.15) | 1.12 (0.81–1.55) | 1.12 (0.81–1.55) | 2.68 (2.04–3.54) | 1.71 (1.18–2.48) | 1.71 (1.18–2.48) |
| Coronary artery disease | 1.43 (1.02–2.00) | 1.15 (0.78–1.70) | 1.16 (0.78–1.70) | 3.35 (2.47–4.55) | 2.05 (1.39–3.02) | 2.05 (1.39–3.02) |
| Osteoporosis | 2.49 (1.60–3.89) | 2.27 (1.39–3.72) | 2.28 (1.39–3.73) | 3.07 (1.96–4.82) | 2.70 (1.54–4.73) | 2.70 (1.54–4.73) |
| Cerebral vascular accident | 1.33 (0.89–1.98) | 1.11 (0.71–1.75) | 1.11 (0.71–1.76) | 1.35 (0.87–2.09) | 0.81 (0.46–1.42) | 0.81 (0.46–1.42) |
| Asthma | 2.82 (1.91–4.16) | 0.83 (0.47–1.44) | 0.83 (0.47–1.44) | 3.40 (2.18–5.30) | 0.41 (0.21–0.78) | 0.41 (0.21–0.79) |
| COPD | 3.56 (2.75–4.62) | 3.26 (2.24–4.73) | 3.26 (2.25–4.74) | 5.83 (4.36–7.78) | 7.07 (4.58–10.91) | 7.07 (4.58–10.90) |
| Chronic kidney disease | 1.23 (0.64–2.36) | 1.16 (0.56–2.40) | 1.16 (0.56–2.40) | 1.95 (1.11–3.40) | 1.01 (0.50–2.06) | 1.01 (0.50–2.06) |
| Chronic liver diseases | 8.94 (7.03–11.38) | 8.30 (6.44–10.70) | 8.26 (6.41–10.65) | 12.28 (9.43–15.99) | 11.01 (8.25–14.69) | 10.98 (8.23–14.65) |
| Hyperthyroidism | 8.00 (3.88–16.50) | 6.12 (2.81–13.32) | 6.12 (2.81–13.32) | 6.33 (3.07–13.05) | 3.88 (1.50–10.04) | 3.88 (1.50–10.03) |
| Thyroiditis | 16.00 (3.40–75.35) | 9.82 (1.70–56.69) | 9.80 (1.70–56.52) | 22.00 (4.88–99.25) | 10.99 (2.18–55.38) | 10.98 (2.18–55.38) |
| ITP | 4.00 (0.25–63.95) | 2.04 (0.12–34.70) | 2.04 (0.12–34.79) | NC | NC | NC |
| AIHA | NC | NC | NC | NC | NC | NC |
| Thrombocytopenia | 13.33 (3.67–48.45) | 14.95 (3.50–63.94) | 14.94 (3.50–63.84) | 12.00 (2.42–59.45) | 6.27 (1.04–37.67) | 6.27 (1.04–37.69) |
| Antiphospholipid syndrome | NC | NC | NC | NC | NC | NC |

Abbreviations: OR: odds ratio; aOR: adjusted odds ratios; CI: confidence intervals; NTDs: New Taiwan dollars; Q, quartile; IQR, interquartile range; DM: dermatomyositis; PM: polymyositis; NA: not available; NC: not calculable.

**Supplementary figure 1. Distributions of months at dengue diagnosis in patients with systemic autoimmune rheumatic diseases (SARDs) and controls**

**
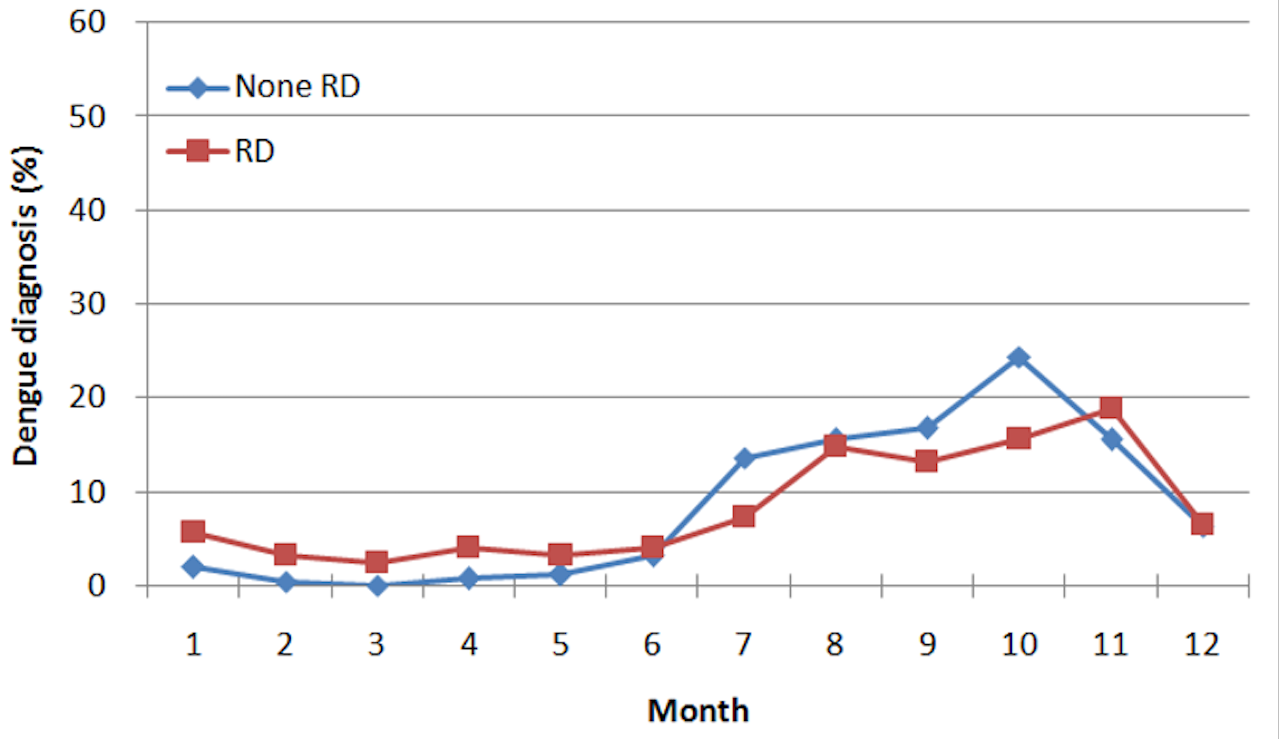
**

**Supplementary figure 2. Distributions of months at dengue diagnosis in patients with systemic lupus erythematosus (SLE) and controls**

**
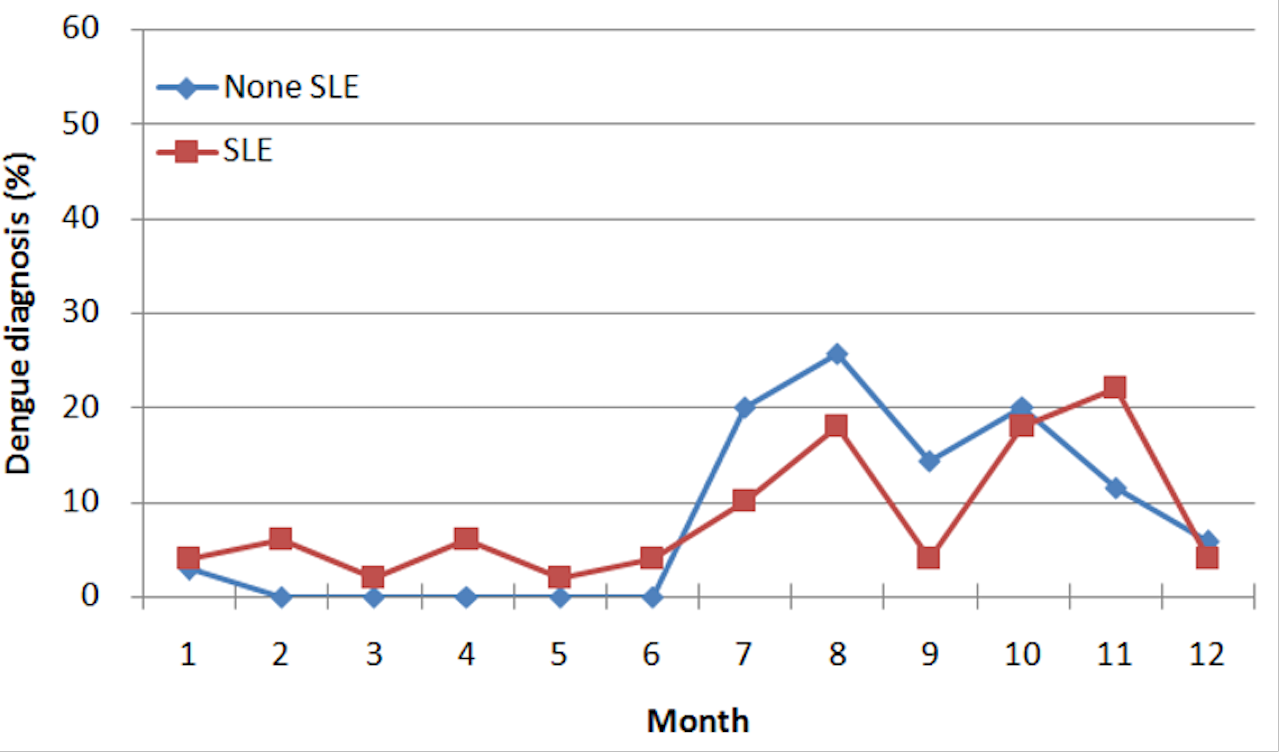
**

**Supplementary figure 3. Distributions of months at dengue diagnosis in patients with Sjogren's syndrome (SS) and controls**

**
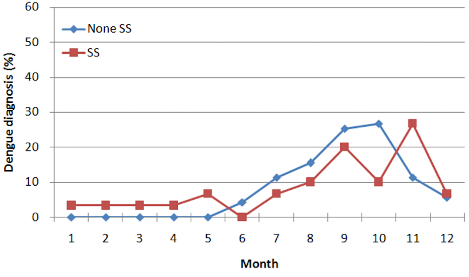
**

**Supplementary figure 4. Distributions of months at dengue diagnosis in patients with rheumatoid arthritis (RA) and controls**

**
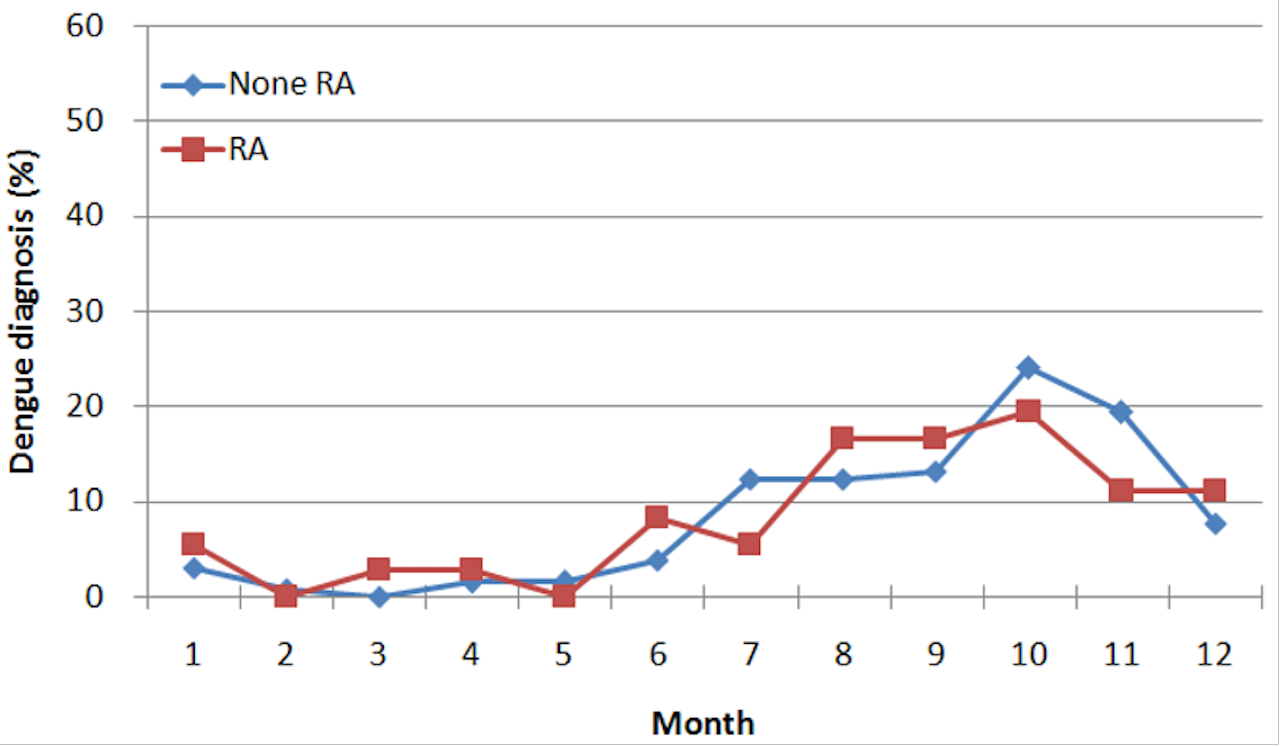
**

**Supplementary figure 5. Distributions of months at dengue diagnosis in patients with systemic sclerosis (SSc) and controls**

**
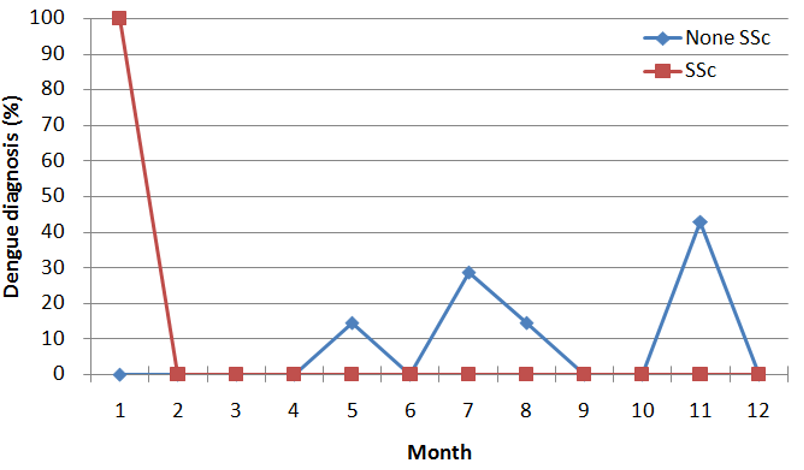
**

**Supplementary figure 6. Distributions of months at dengue diagnosis in patients with dermatomyositis (DM) and controls**

**
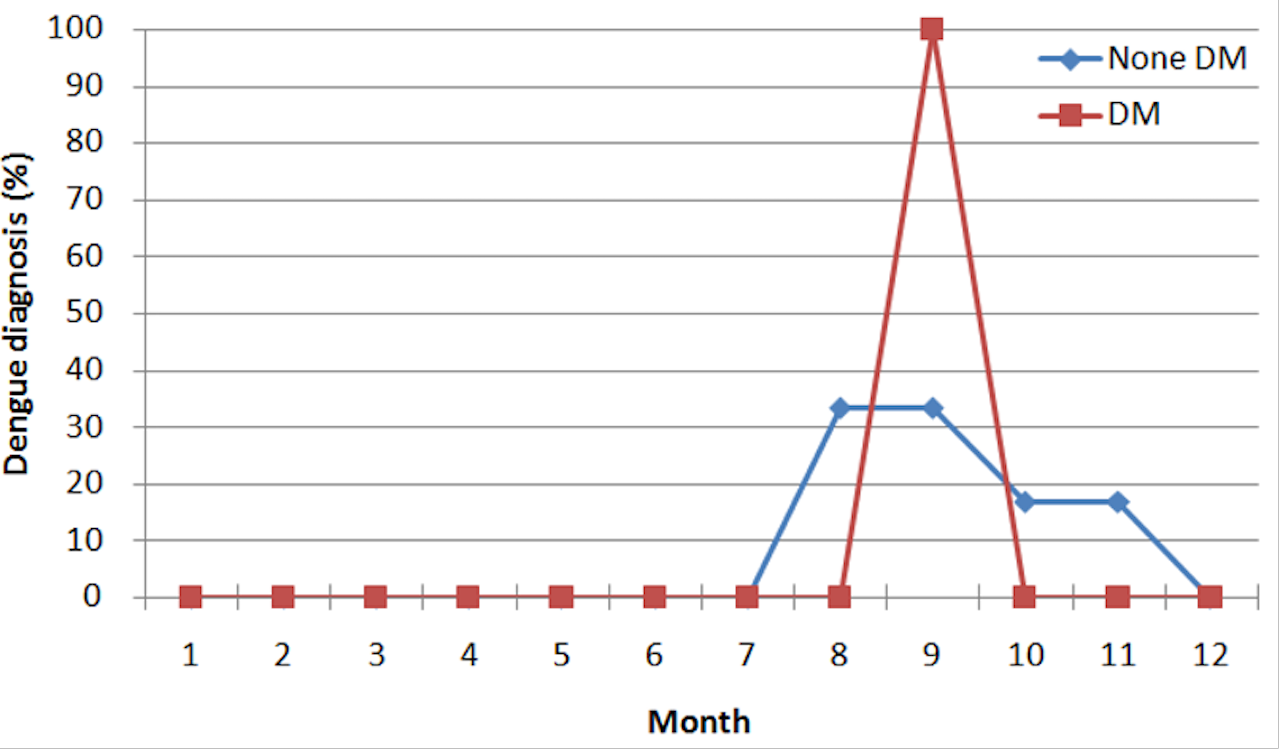
**

**Supplementary figure 7. Distributions of month at dengue diagnosis in patients with polymyositis (PM) and controls**

**
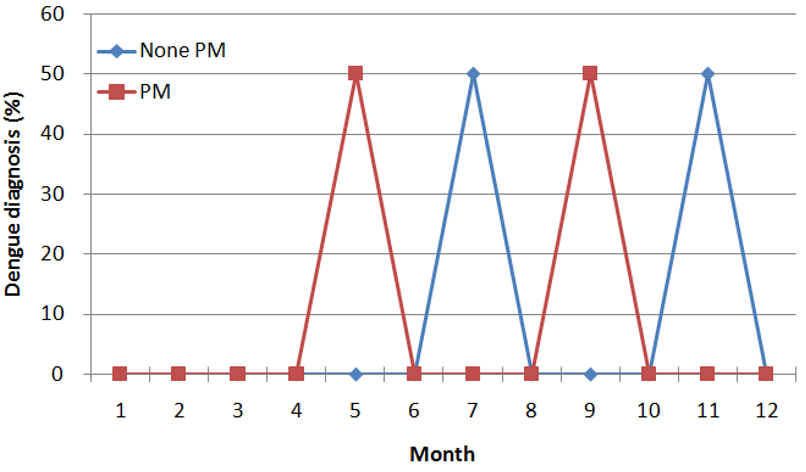
**
